# Supplementary material for: The role of project management office in the implementation of strategic plans in project-based organisations
Source: PLoS One. 2024 Jul 18;19(7):e0306702. doi: 10.1371/journal.pone.0306702 (PMC11257362; doi:10.1371/journal.pone.0306702)
Supplement: Attachment — Submitted filename: PONE-D-24-08102.docx [file pone.0306702.s002.docx]

The manuscript titled "The Role of PMO Practices in the Implementation of Strategic Plans in Project Based Organisations" has been critically reviewed. I have some minor suggestions that can enhance the manuscript. It presents a thorough investigation into the impact of Project Management Office (PMO) roles on strategic plan implementation within project-based organizations. I have so many major mistake in this manuscript. It not look like a research paper, because the authors didn’t follow the proper scholarly write-up format. To further enhance the quality and impact of this research work, I have the following comments and suggestions:

**Title:** The title of paper is very confusing. No need to use Abbreviation in the title.

**Abstract:** Abstract doesn’t contain relevant information but it contain a lot of extra information which should not be a part of paper abstract.

**Clarify the Research Gap**: The introduction clearly identifies a gap in the existing literature regarding the strategic role of PMOs. To strengthen this further, it would be beneficial to explicitly state how your study's findings fill this gap and what unique contributions your research makes to the field.

**Expand Literature Review**: While the literature review is comprehensive, incorporating recent studies could enrich the context. Highlighting studies conducted after 2020 might provide a more current understanding of PMO roles and their evolution in response to emerging project management challenges.

**Methodology Detailing**: The methodology section is well-structured, but adding more details about the survey design and statistical analysis techniques (beyond regression analysis) could enhance reproducibility and the rigor of your study. Discussing the rationale for choosing the specific quantitative methods and any limitations they present would also be valuable.

**Sample**: Your sample is robust and geographically diverse, which strengthens the study. However, exploring the implications of PMO practices across different industries or sectors more explicitly could provide deeper insights into how context influences PMO effectiveness in strategic plan implementation.

**In-depth Analysis of Non-significant Roles**: The results section provides a clear distinction between significant and non-significant PMO roles. An in-depth discussion on why certain roles (e.g., Organisational Learning Promotion) were found to be non-significant and the potential implications for practice could offer additional value to readers.

**Practical Implications**: The discussion on practical implications is insightful but could be expanded. Providing specific recommendations for project managers and PMO leaders on how to apply your findings in their organizational contexts would make the research more actionable.

**Future Research Directions**: While the conclusion mentions the potential for future research, outlining specific questions or areas of investigation that emerged from your findings could guide subsequent studies and encourage further exploration into PMO roles and strategic planning.

**Theoretical Contributions**: Elaborate more on how your findings contribute to or challenge existing theories within project management and strategic planning literature. This would enhance the theoretical significance of your study.

**Interdisciplinary Insights**: Considering the interdisciplinary nature of PMOs, drawing connections to related fields such as organizational behavior, information systems, and strategic management could enrich the discussion and appeal to a broader audience.

**Enhance Visuals and Tables**: The figures and tables are informative but ensuring they are as clear and concise as possible will help readers digest the information more effectively. Consider revising to focus on key findings and using supplementary materials for additional details.

Implementing these suggestions could enhance the clarity, depth, and impact of your research, making a valuable contribution to the understanding of PMO roles in strategic plan implementation within project-based organizations.
