# Supplementary material for: The role of project management office in the implementation of strategic plans in project-based organisations
Source: PLoS One. 2024 Jul 18;19(7):e0306702. doi: 10.1371/journal.pone.0306702 (PMC11257362; doi:10.1371/journal.pone.0306702)
Supplement: Attachment — Submitted filename: Reviewers comments.docx [file pone.0306702.s003.docx]

**Introduction**

- Some points are repeated across different paragraphs, such as the discussion on the challenges faced by project-based organizations and the role of PMO. This repetition can make the text redundant and less engaging.
- Certain statements are vague and could benefit from more specific examples or evidence. For instance, phrases like "unfavourable challenges" and "effective approaches and tools" lack specificity and clarity.

**Background**

- Some sentences are lengthy and could be simplified for better clarity and readability. For example, the sentence starting with "Many research studies stated..." could be broken down into smaller, clearer sentences.
- Could you please correct the usage of 'PMO' as an abbreviation in two instances: firstly, in the introduction and secondly, in the section discussing the importance of PMO for an organization? Additionally, on line 152, ensure that 'project management office' is spelled out in full, with its abbreviation included. Correct and put abbreviation.
- On page 10, line 216, tackled and gained on" might be more effectively expressed as "addressed and acquired."
- Utilize Strategic human resource management practitioners’ emotional intelligence and affective organizational commitment in higher education institutions in Georgia during post-COVID-19(2023) to enhance your discussion on strategic management. It offers valuable insights on Conceptualization of SM strengthening your argument regarding strategic management.

**methodology**

- It would be beneficial to provide some insight into the process of composing the questionnaire contents to demonstrate the thoroughness of the methodology.

Other suggestions:

### This study lacks the exploration of both theoretical and practical implications. Please add.

- Please incorporate considerations of limitations and suggest avenues for further research.
